# MENTAL HEALTH AND FINANCIAL HARDSHIP AMONG OLDER ADULTS WITH PROBABLE COVID-19 INFECTION

**DOI:** 10.1093/geroni/igac059.761

**Published:** 2022-12-20

**Authors:** Paola Zaninotto, Eleonora Iob, Andrew Steptoe

**Affiliations:** UCL, London, England, United Kingdom; UCL, London, England, United Kingdom; University College London, London, England, United Kingdom

## Abstract

We investigated the immediate and longer-term impact of probable COVID-19 infection on mental health, wellbeing, and financial hardship among older people living in England. Data were analysed from 5146 older adults participating in the English Longitudinal Study of Ageing who provided data before the pandemic (2018-19) and at two COVID-19 assessments in 2020 (June-July and November-December). The associations of probable COVID-19 infection (first COVID-19 assessment) with depression, anxiety, poor quality of life (QoL), loneliness, and financial hardship at the first and second COVID-19 assessments were tested using linear/logistic regression and were adjusted for pre-pandemic outcome measures. Participants with probable infection had higher levels of depression and anxiety, poorer QoL, and greater loneliness scores compared with those without probable infection at both the first (ORdepression=1·62[95%CI:1·16,2·26];ORanxiety=1·59[95%CI:1·00,2·51]; bpoorQoL=1·34[95%CI:0·66,2·02]; bloneliness=0·49[95%CI:0·25,0·74]) and second (ORdepression =1·56[95%CI:1·17,2·09];ORanxiety=1·55[95%CI:1·02,2·37];bpoorQoL=1·38[95%CI:0·74,2·03]; bloneliness=0·31[95%CI:0·04;0·58]) assessments. Participants with probable infection also experienced greater financial difficulties than those without infection at the first assessment (OR=1·50[95%CI:1·10,2·05]).

